# Variations in conventional and non-conventional semen characteristics of selected rabbit breeds

**DOI:** 10.1080/10495398.2025.2548300

**Published:** 2025-08-22

**Authors:** Eva Tvrdá, Filip Benko, Michal Ďuračka, Anton Kováčik, Jakub Vozaf, Andrea Svoradová, Jaromír Vašíček, Andrej Baláži, Peter Supuka, Simona Kunová, Jana Žiarovská, Miroslava Kačániová, Peter Chrenek

**Affiliations:** ^a^ Institute of Biotechnology, Faculty of Biotechnology and Food Sciences, Slovak University of Agriculture in Nitra, Nitra, Slovakia; ^b^ Chair of Animal Breeding and Biotechnology, Institute of Veterinary Medicine and Animal Sciences, Estonian University of Life Sciences, Tartu, Estonia; ^c^ AgroBioTech Research Centre, Slovak University of Agriculture in Nitra, Nitra, Slovakia; ^d^ Institute of Applied Biology, Faculty of Biotechnology and Food Sciences, Slovak University of Agriculture in Nitra, Nitra, Slovakia; ^e^ NPPC, Research Institute for Animal Production Nitra, Lužianky, Slovakia; ^f^ Department of Morphology, Physiology and Animal Genetics, Faculty of AgriSciences, Mendel University in Brno, Brno, Czechia; ^g^ Vetservis, s.r.o, Nitra, Slovakia; ^h^ Institute of Food Sciences, Faculty of Biotechnology and Food Sciences, Slovak University of Agriculture in Nitra, Nitra, Slovakia; ^i^ Institute of Plant and Environmental Sciences, Faculty of Agrobiology and Food Resources, Slovak University of Agriculture in Nitra, Nitra, Slovakia; ^j^ Institute of Horticulture, Faculty of Horticulture and Landscape Engineering, Slovak University of Agriculture, Nitra, Slovakia; ^k^ School of Medical and Health Sciences, University of Economics and Human Sciences in Warsaw, Warsaw, Poland

**Keywords:** Sperm quality, semen quality, rabbit breeds

## Abstract

This article strove to characterize and compare biochemical, oxidative, bacteriological and immunological semen properties in Zemplin, Liptov Baldspotted and New Zealand rabbits. Besides, bacteria were characterized using the matrix-assisted laser desorption/ionization time-of-flight mass spectrometry. Oxidative profile of semen was assessed using chemiluminescent and colorimetric protocols. Levels of pro-inflammatory cytokines were evaluated with the enzyme-linked immunosorbent assay. Seminal plasma biochemistry was assessed with an automated clinical chemistry analyser. The lowest sperm concentration and motility were found in the Liptov Baldspotted ejaculates which also presented with significantly (*p* < 0.01) elevated levels of tumour necrosis factor alpha (TNF-α) and interleukin 1, free radicals (*p* < 0.0001) and malondialdehyde (*p* < 0.0001) in comparison to New Zealand rabbits. The prevailing bacterial genera in semen were *Stenotrophomonas* spp., *Staphylococcus* spp., *Micrococcus* spp. and *Acinetobacter* spp. Significantly increased levels of alanine transaminase and creatinine (Crea) were found in New Zealand rabbits in comparison to the Liptov Baldspotted breed (*p* < 0.001). Overall, Liptov Baldspotted rabbits produced semen of lower quality than Zemplin and New Zealand rabbits, suggesting that this breed may be more predisposed to a higher susceptibility to internal and external stresses which may interfere with male fertility.

## Introduction

In the forthcoming decades, the demand for livestock products will increase proportionately to the rise in the world population in addition to consumers becoming more conscious and demanding high-quality animal goods.^1^ In this sense, rabbits represent a valuable alternative source of highly nutritious and easily digestible meat with low fat and cholesterol.^2^ As rabbits are being chosen as livestock because of their reproductive and feeding advantages, lower labour and cost requirements, modern rabbit breeding needs to face important challenges for it to remain a profitable, competitive and sustainable sector.^3^

Fertility is the first and foremost aspect to be taken into consideration in rabbit breeding, since the litter size determines the production sustainability and profitability. While a traditional approach to maintain a colony relies on repeated breeding, severe inbreeding depression is a common phenomenon observed in rabbits,^4^^,^^5^ hence a significant number of bucks is needed for mating, incrementing the production costs. As such, the attention of breeders has gradually shifted towards cryopreservation, which, if properly executed, offers in theory a semi-eternal preservation of spermatozoa in liquid nitrogen to maintain their fertility.^4^

Bucks represent the foundation of rabbit breeding programs, since a single male may fertilize numerous does when artificial insemination (AI) is performed.^4^ Correspondingly, the success of rabbit AI programs depends primarily on male health and reproductive performance at the time of semen collection. The quality may be highly variable between collections^6^ and easily affected by a wide variety of both internal and external factors, such as age, breed, health status, housing conditions or feed regimen.^7^

Semen quantity and/or quality is generally assessed by a wide domain of traits, including qualitative parameters of the ejaculate, biochemical composition of the specimen and cell vitality.^8^ Most reports on the relationship between fertility and semen properties in rabbits have only considered sperm concentration or motility as the principal attributes that may indirectly define fertility.^8^^,^^9^ Nevertheless, it is clear now that the ejaculate is a heterogenous fluid comprising a complex network of molecules with multiple roles in the sperm transport, motility enhancement, stimulation of capacitation or hyperactivation, maintenance of sperm viability and protection of male gametes against oxidative stress.^10^^,^^11^

Besides motility-promoting, signalling, antioxidant and immune molecules, semen has been recently shown to host a plethora of microorganisms that may originate from the urogenital system or preputial fluids, as well as from the skin, fur, urine, or feces.^12^ It is now acknowledged that bacteriospermia accompanied by inflammatory processes may be associated with the loss of sperm structural integrity and functional activity, reactive oxygen species (ROS) overproduction and cell death, leading to sub-standard semen quality even in clinically healthy studs.^13–17^

While currently available studies on male reproduction in rabbits have primarily addressed changes in the sperm quality affected by age,^18^ diet^19^ or seasonality,^20^ the impact of a complex set of variations in selected conventional, and molecular indicators of sperm structural integrity and functional activity is still missing. This study employed a comprehensive comparative approach to investigate the ejaculates of the Zemplín rabbit and the Liptov Baldspotted rabbit, two esteemed national rabbit breeds of Slovakia renowned for their genetic and cultural significance.^21^ These breeds were selected due to their unique characteristics and importance to biodiversity conservation efforts. To provide a benchmark for evaluation, New Zealand rabbits, a widely recognized and extensively studied breed with less health problems in comparison with other breeds,^22^ were included as a control group. This approach allowed for a detailed assessment of breed-specific traits and potential implications for breeding strategies and genetic resource preservation.

## Materials and methods

### Bioethics and animals

The animals and sample collections were carefully handled in accordance with the ethical guidelines stated in the Slovak Animal Protection Regulation RD 377/12, which conforms to European Union Regulation 2010/63. Since semen collection is routinely performed at the Research Institute for Animal Production Nitra, causing no harm or discomfort, special ethical approval was not needed for this type of experiment.

Semen samples were collected from clinically healthy and sexually mature rabbits of the Zemplin (*n* = 10), Liptov Baldspotted (*n* = 10) and New Zealand (*n* = 10) rabbit breed over the period of two months. The animals were 1–1.5 years old, with an average weight of 3.5 ± 4.5 kg and kept at the experimental farm of the Research Institute for Animal Production Nitra (Lužianky, Slovak Republic) since birth. The rabbits were housed in a partially air-conditioned rabbit house under a photoperiod of 16 L:8D, kept in individual cages and fed with a commercial diet. Water was provided *ad libitum*. Air temperature of 20–24 °C and relative humidity of 65% were maintained in the rabbit house.^23^^,^^24^ The animals underwent regular health checkups by a veterinarian, focusing primarily on any nose, eye or ear abnormalities or discharge; mouth and fur for signs of disease; as well as rear skin for urine staining or stuck faeces.

### Semen collection

Three ejaculates were obtained from each rabbit using an artificial vagina. To minimize the risk of bacterial contamination during semen collection, strict hygienic protocols were followed. The artificial vagina was thoroughly sterilized with boiling water and antibacterial soap between each use, collection was performed in a sanitized laboratory environment, and personnel changed sterile gloves between handling each animal. Subsequently, the samples were transported to the AgroBioTech Research Center using a thermal vessel at 37 °C (M&G Int., Renate, Italy), and split into three aliquots.

The first aliquot of native semen was pipetted into a sterile Eppendorf tube in a sterilized laminar flow cabinet and immediately frozen at −80 °C for further bacteriological analysis.

The second aliquot was diluted in sterile PBS (phosphate-buffered saline without Ca^2+^ or Mg^2+^; Sigma-Aldrich, St. Louis, MO) at a 1:10 ratio for the assessment of conventional sperm characteristics, leukocyte concentration and ROS generation.

The third aliquot was centrifuged (2300 × *g*, 20 °C, 5 min), to obtain the seminal plasma which was then stored at −80 °C for the assessment of the total antioxidant status (TAS) and selected pro-inflammatory molecules as well as for clinical biochemistry assays. The sperm fraction obtained by centrifugation was treated with the radioimmunoprecipitation assay (RIPA) buffer with protease inhibitor cocktail (Sigma-Aldrich), lysed on ice with an ultrasonic homogenizer (28 kHz) and centrifuged (2300 × *g*, 4 °C, 5 min). The obtained lysates were subjected to the quantification of proteins using the Total protein assay (Randox Laboratories, Crumlin, UK) and the Monza photometric analyser (Randox Laboratories), and then stored at −80 °C for the assessment of oxidative damage to the proteins and lipids and for the Western blots.^25^

### Conventional sperm parameters

Sperm concentration and motility were assessed with computer-assisted sperm analysis (CASA; Version 14.0 TOX IVOS II.; Hamilton–Thorne Biosciences, Beverly, MA). The setup was adjusted to the following cut-off values: frames acquired: 30; frame rate: 60 Hz; minimum contrast: 50; minimum cell size: 7 pixels; minimum static contrast: 30; cell size: 5 pixels; cell intensity: 70; static head size: 0.80–4.93; static head intensity: 0.49–1.68; static elongation: 22–84. In order to determine the sperm motion behaviour without the interference of detritus, the specimens were stained with the IDENT stain (Hamilton-Thorne Biosciences) and analysed under fluorescent illumination at 37 °C. Makler counting chamber (depth 10 µm; Sefi Medical Instruments, Haifa, Israel) was used to load the specimen into the CASA system, and a minimum of 300 spermatozoa were evaluated per each run. Sperm concentration is expressed as 10^6^ spermatozoa/mL, while the motility is defined as the percentage of spermatozoa moving faster than 5 μm/s.^23^

For the assessment of sperm membrane integrity and the occurrence of dead spermatozoa, 1 × 10^6^ cells were stained with 10 μL CFDA (carboxyfluorescein diacetate; Sigma-Aldrich; 0.75 mg/mL in DMSO), 10 μL DAPI (4′,6-diamidino-2-phenylindole; Sigma-Aldrich; 1 μM in PBS), 10 μL PI (propidium iodide; Sigma-Aldrich; 1 μM in PBS) and incubated at 37 °C for 15 min. The principle of CFDA staining lies in its ability to cellular esterase activity, indicative of cell viability. Once this dye enters the cell, esterases will actively cleave its acetoxymethyl groups, leading to the production of fluorescent 5-carboxyfluorescein that will remain trapped intracellularly, indicating that the cell is viable since only intact membranes can support the intracellular environment crucial for esterase activity. In the meantime, PI will identify cells affected by late apoptosis or necrosis while the nucleic acid dye DAPI will indicate the total number of spermatozoa.^26^ Following incubation, the samples were centrifuged (150 × *g*, 5 min), washed with 100 µL PBS twice and resuspended in 100 µL PBS. At least 300 cells were counted under an epifluorescence microscope with a × 40 magnification (Leica Microsystems, Wetzlar, Germany). CFDA-positive cells were classified as membrane-intact (%) while PI-positive cells were evaluated as dead.^25^

For the acrosome integrity, 1 × 10^6^ cells were stained with 100 μL PNA (peanut agglutinin, FITC conjugate; Sigma-Aldrich; 10 μM in PBS) and 10 μL DAPI. The staining procedure was carried out without a permeabilization step, resulting in PNA binding to and faintly staining the surface of acrosome-reacted spermatozoa. As such, PNA-positive cells were acknowledged as acrosome-reacted.^27^ The specimens were incubated at 37 °C for 15 min and analysed with an epifluorescence microscope (×40 magnification; Leica Microsystems). A minimum of 300 cells were counted, and PNA-negative cells were classified as acrosome-intact (%).^25^

Mitochondrial activity was evaluated with the JC-1 Mitochondrial Membrane Potential Assay kit (Cayman Chemical, Ann Arbor, MI). The JC-1 fluorescent dye (5.5′,6.6′-tetrachloro-1,1′,3,3′-tetraethylbenzimidazolylcarbocyanine iodide) was diluted in PBS and 5 μL of the JC-1 solution were mixed with 1 × 10^6^ cells adjusted to 100 μL and incubated at 37 °C for 30 min. The specimens were centrifuged (150 × *g*, 5 min) and washed twice with a JC-1 washing buffer. All samples were then analysed with the GloMax-Multi^+^ spectro-fluoro-luminometer (Promega, Madison, WI). Mitochondrial membrane potential is expressed as the red/green ratio (ratio of JC-1 monomers to JC-1 complexes).^23^

Sperm DNA fragmentation index (%) was assessed with the Halomax kit (Halotech DNA, Madrid, Spain). A small aliquot (20 μL) of each specimen was fixed on agarose-covered slides, treated with a lysis buffer (5 min), distilled water (5 min), ethanol (70% and 100%; 2 min each) and air-dried. The slides were stained with SYBR Green (2 μg/mL DMSO) (Sigma-Aldrich) and Vectashield (Vector Laboratories, Burlingame, CA) and at least 300 cells were evaluated under an epifluorescence microscope (Leica Microsystems, Wetzlar, Germany) using a × 40 magnification objective.^23^

### Oxidative profile

ROS production was quantified with luminol (Sigma-Aldrich)–based chemiluminescent protocol as previously described.^23^^,^^25^ This assay is based on the production of light *via* interactions between luminol, intracellular as well as extracellular ROS. The emitted light signal is then converted to an electrical signal by a luminometer.^28^ Hydrogen peroxide (H_2_O_2_; 30%; 8.8 M; Sigma-Aldrich) served as a positive control for the assay. The resulting luminescent signal was recorded in fifteen 1-min cycles with the Glomax Multi^+^ combined spectro-fluoro-luminometer. The extent of ROS production is expressed in relative light units (RLU)/s/10^6^ sperm.

Total antioxidant capacity as the overall sum of molecules with antioxidant properties in the sample was assessed in the seminal plasma aliquots using the improved chemiluminescence assay employing a signal reagent composed of luminol, 4-iodophenol (Sigma-Aldrich), horseradish peroxidase (Sigma-Aldrich) and H_2_O_2_, which create a cell-free oxygen radical generating system that will be counteracted by the antioxidants. The reaction occurs in the presence of a peroxidase enzyme stabilizer (StabilZyme^®^ stabilizer; Surmodics IVD, Eden Prairie, MN) to prolong ROS generation and hence monitor the reaction at a steady rate.^29^ The light signal emitted by the reaction was recorded during 10 consecutive one-minute-long cycles with the Glomax Multi^+^ spectro-fluoro-luminometer. The result was calibrated with reference to Trolox (5–100 μmol/L; 6-hydroxy-2,5,7,8-tetramethylchroman-2-carboxylic acid; Sigma-Aldrich) and expressed as μmol Trolox Eq./g protein.^25^

Oxidative damage to the proteins expressed through the amount of protein carbonyls (PC) was assessed with a modified DNPH (dinitrophenylhydrazine) method.^30^ The protocol is based on the reaction of DNPH with the carbonyls of previously pelleted proteins with 1 mL of trichloroacetic acid (TCA; Sigma-Aldrich), leading to the formation of a protein carbonyl-DNPH hydrazone. The samples were treated with 1 mL TCA again, cooled down and centrifuged (805 × *g*, 5 min). The pellet was washed 3 times with 500 µL ethanol/ethyl acetate (Sigma-Aldrich) to remove free DNPH that exhibits peak absorbance at the same wavelength as carbonyl-DNPH hydrazones. The resulting pellet was resuspended in 1 mL 6 M guanidine hydrochloride (Sigma-Aldrich) and subjected to spectrophotometric measurement at 360 nm with the Cary UV–VIS spectrophotometer (Cary Systems, Santa Clara, CA). Carbonyl-DNPH hydrazone groups were quantified by the DNPH absorption extinction coefficient 22,000 M^−1 ^cm^−1^. Oxidative damage to the proteins is expressed in nmol PC/mg protein.^25^

Thiobarbituric acid-reactive substances (TBARS) assay was used to quantify malondialdehyde (MDA) as the prime product of oxidative damage to lipids. The principle of the assay stems from the interactions of MDA with thiobarbituric acid in the presence of heat and acidic conditions to produce a coloured end-product that absorbs light at 530–540 nm. Subsequently, the intensity of the colour reaction corresponds to the level of lipid peroxidation in the sample. The lysates (100 μL) were treated with 4 mL 0.53% thiobarbituric acid (Sigma-Aldrich) in 20% acetic acid (pH 3.5; Centralchem, Bratislava, Slovakia) and exposed to 100 °C for 60 min. The samples were then cooled down for 10 min, centrifuged (1300 × *g*, 10 min). The supernatants were processed with the Glomax Multi^+^ spectro-fluoro-luminometer at a wavelength of 540 nm. MDA levels were calculated using a standardization curve. The results are quoted as µmol MDA/g protein.^25^

### Inflammatory profile

Leukocyte quantity was assessed with the Endtz test. Twenty µL of diluted ejaculates (1:10) were treated with 40 µL of the Endtz working solution^25^ and incubated at 20 °C for 5 min. Stained leukocytes were quantified under a bright-field microscope (×1000; Nikon ECLIPSE E100, Tokyo, Japan). The results are expressed as ×10^6^ leukocytes/mL.

Commercially available ELISA kits designed for samples of rabbit were used to quantify interleukin 1 beta (IL-1β), interleukin 6 (IL-6), interferon gamma (IFN-γ) (Cat. no. # ER5RBX5, # EH2IL6, # ER4RB; Thermo Fisher Scientific, Waltham, MA), tumour necrosis factor alpha (TNF-α) and C-reactive protein (CRP) (Cat. no. # MBS2500169, # MBS724197; MyBioSource Inc., San Diego, CA). All assays followed double-sandwich ELISA protocol and the resulting absorbances were measured with the help of the Glomax Multi^+^ spectro-fluoro-luminometer at 450 nm.

### Western blot

Three randomly selected sperm lysates with a suitable protein concentration were selected from each group for the Western blot analysis of the pro-apoptotic BAX protein and the anti-apoptotic Bcl-2 protein. Prior to the assay, the protein concentration of the samples was adjusted using PBS to reach a final concentration of 25 μg protein. The samples were then treated with 4× Laemli buffer (BioRad, Hercules, CA) and β-mercaptoethanol (Sigma-Aldrich), and subsequently boiled at 95 °C for 10 min. The samples were loaded (20 μL) into Mini-PROTEAN TGX stain-free polyacrylamide gels (BioRad), together with 7 μL of Precision Plus Protein marker (BioRad). SDS-polyacrylamide gel electrophoresis was run at 90 V for 2 h, and the gels were visualized with the ChemiDoc Imaging System (BioRad) to confirm the loading uniformity. The gels were then transferred to polyvinylidene difluoride membranes (Trans-Blot Turbo Pack; BioRad) using the Trans-Blot Turbo Transfer System (BioRad) at 25 V and 2.5 A, for 7 min. The blotting sandwich was disassembled, and the membranes were incubated for 3 × 10 min in Tris-buffered saline (TBS) composed of Tris base (Sigma-Aldrich), sodium chloride (Centralchem) and UHQ water. This step was followed by membrane staining with Ponceau S solution (Sigma-Aldrich) to visualize the bands on the membranes. The membranes were blocked with 5% skim milk (Blotting grade blocker; BioRad) in TBS containing 0.1% Tween-20 (Sigma-Aldrich). Membrane blocking was performed on a stirrer at room temperature for 2 h. Finally, the membranes were incubated with one of the following primary antibodies:

- Bax Polyclonal Antibody (# BS-0127R; Bioss Antibodies Inc.); 1:1 000 in 5% milk in TBS/0.1% Tween-20.
- Bcl-2 Polyclonal Antibody (# BS-0032R; Bioss Antibodies Inc.); 1:1 000 in 5% milk in TBS/0.1% Tween-20.

The next day, the membranes were washed for 5 × 10 min in a wash buffer composed of 1% milk in TBS/0.2% Tween-20 and subsequently incubated with a secondary antibody (GE Healthcare, Chicago, IL) diluted 1:15000 in 1% milk in TBS/0.2% Tween-20 for 1 h. Following incubation, the membranes were washed for 3 × 10 min in TBS/0.2% Tween-20 at room temperature and using a stirrer. To visualize the target protein, membranes were incubated with the ECL substrate (GE Healthcare) in the dark for 5 min. After incubation, the membranes were placed into the ChemiDoc Imaging System, which automatically calculated the protein visualization time based on the light signal emitted by the membranes.^31^

### Seminal plasma biochemistry

Since the seminal plasma biochemistry plays important roles in the process of sperm production, maturation and metabolism, selected biochemical components of the seminal plasma including calcium (Ca), phosphorus (P), magnesium (Mg), urea (U), total proteins (TP), albumin (Alb), alanine aminotransferase (ALT), cholesterol (Chol), triglycerides (TAG), uric acid (UA) and creatinine (Crea) were measured using commercial kits purchased from Randox Laboratories on the fully automated clinical chemistry analyser RX Monaco (Randox Laboratories).^32^

### Bacterial cultures

For the identification of the bacterial species in rabbit semen, 100 µL of each specimen was inoculated onto a sterile blood agar (blood agar base no. 2), Gassner agar (NutriSelect^®^ basic) and tryptone soy agar (soybean casein digest agar) (Merck, Darmstadt, Germany). The plates were then incubated under aerobic conditions at 36 ± 2 °C for 24 h. Subsequently, the colonies were counted and transferred to a fresh tryptone soya aga to obtain pure cultures, which were incubated again under aerobic conditions at 37 ± 1 °C for 24 h.^23^

### Bacteriological identification

The isolated and purified bacterial cultures were identified using MALDI-TOF Biotyper mass spectrometry (Brucker Daltonics, Bremen, Germany). A small amount of a purified culture was mixed with 300 μL distilled water. Subsequently, 900 μL 99.8% ethanol (Centralchem) was added and the mixture was centrifuged (750 × *g*, 2 min). The resulting pellet was allowed to dry freely and then resuspended thoroughly in 30 μL 70% formic acid (Sigma-Aldrich) and 30 μL acetonitrile (Sigma-Aldrich). The samples were subsequently centrifuged at 805 × *g* at room temperature for 2 min. One μL of the supernatant was placed on the MALDI identification plate, allowed to dry freely and subsequently covered with a working solution of MALDI matrix composed of acetonitrile, ultrapure water, trifluoroacetic acid and cinnamic acid (Sigma-Aldrich). Identification of the bacterial isolates was performed using the Microflex LT instrument with the flexControl software version 3.4 (Brucker Daltonics, Bremen, Germany). The spectra obtained by the mass spectrometry were compared with the MALDI Biotyper Bruker Taxonomy database (Bruker Daltonics).^23^

### Antibiotic resistance testing

Randomly selected bacterial isolates were furthermore tested for antibiotic resistance. The antimicrobial susceptibility test was executed with the disk diffusion method against 30 mg amikacin, 30 mg cefepime, 30 mg chloramphenicol, 10 mg ciprofloxacin, 10 mg doripenem, 10 mg gentamicin, 10 mg imipenem, 10 mg linezolid, 10 mg meropenem, 30 mg tetracycline, 15 mg tigecycline, 10 mg tobramycin and 30 mg vancomycin according to Kačániová et al.^33^

### Biodiversity calculation

Descriptive analysis of bacterial occurrence in individual rabbit breeds was prepared by MS Excel as well as total number of species obtained in the individual analysed groups and standard basic diversity parameters. Beside standard indices, Berger–Parker Index was calculated based on the formula **d = max(pi)** to characterize real unbalanced groups different in analysed groups.^25^

### Statistical analysis

GraphPad Prism statistical program version 8.4.4 for Mac (GraphPad Software Incorporated, La Jolla, CA) was used for the analysis. Results are expressed as mean ± standard deviation (S.D.). The samples were divided according to the breed and processed with the Shapiro–Wilk normality test with the data sets passing with non-significant results at the alpha level of 0.05. Differences among the pre-established groups were evaluated by one-way ANOVA and followed by the Tukey multiple comparison test. Levels of statistical significance for both statistical operations were set at: *p < 0.05; ^∗∗^*p* < 0.01; ^∗∗∗^*p* < 0.001; ^∗∗∗∗^*p* < 0.0001.

## Results

### Conventional sperm quality parameters

The data analysis revealed that the sperm concentration was significantly (*p* < 0.0001) decreased in the Zemplin as well as the Liptov Baldspotted breed in comparison to New Zealand rabbits (Table 1). The lowest sperm motility was found in semen collected from Liptov Baldspotted rabbits, which was significantly different in comparison to the Zemplin (*p* < 0.05) as well as New Zealand breed (*p* < 0.0001). Significant differences in the motility were also detected among Zemplin and New Zealand rabbits (*p* < 0.05).

**Table 1.** Conventional sperm quality characteristics amongst three rabbit breeds.

Quality groups	Zemplin	Liptov Baldspotted	New Zealand
Sperm concentration [×10^6^ sperm]	483.20 ± 19.03	511.70 ± 16.78	1126.00 ± 35.20^****Z; ****LB^
Sperm motility [%]	57.98 ± 3.44	44.04 ± 3.61^*Z^	70.80 ± 3.09^*Z; ***LB^
Membrane integrity [%]	77.30 ± 4.36	65.30 ± 3.12	87.20 ± 2.95^**LB^
Dead spermatozoa [%]	15.32 ± 2.41	21.48 ± 3.55^*Z^	11.95 ± 2.15^***LB^
Acrosome integrity [%]	86.20 ± 3.19	72.00 ± 8.08^**Z^	90.60 ± 4.45^**LB^
Mitochondrial membrane potential [JC-1 units]	2.51 ± 0.19	1.32 ± 0.17^*Z^	3.18 ± 0.33^***LB^
Sperm DNA fragmentation [%]	5.84 ± 0.65	6.95 ± 0.58	4.11 ± 0.54^*Z; **LB^

Mean ± S.D. *p < 0.05; **p < 0.01; ***p < 0.001; ^****^p < 0.0001.

^Z^–*vs.* Zemplin rabbits; ^LB^–*vs.* Liptov Baldspotted rabbits. All analyses were run in triplicate.

The assessment of the sperm structural integrity revealed a significantly lower proportion of spermatozoa with intact membranes in Liptov Baldspotted rabbit semen in comparison to the New Zealand breed (*p* < 0.01). Liptov Baldspotted spermatozoa also presented with the lowest percentage of cells with an intact acrosome, which was significantly different in comparison with Zemplin (*p* < 0.01) as well as New Zealand rabbits (*p* < 0.001) as well as with a significantly higher occurrence of dead spermatozoa when compared with both groups of breeds (*p* < 0.05 with respect to the Zemplin breed; *p* < 0.001 in case of the New Zealand breed. The mitochondrial membrane potential was significantly reduced in the Liptov Baldspotted group when compared to Zemplin (*p* < 0.05) as well as New Zealand rabbits (*p* < 0.001). Finally, a significantly lower percentage of spermatozoa with fragmented DNA was observed in New Zealand rabbit semen, in comparison to the Zemplin (*p* < 0.05) and Liptov Baldspotted breed (*p* < 0.01).

### Oxidative profile

With respect to the assessment of the oxidative profile we recorded a significantly higher ROS production in the samples collected from Liptov Baldspotted rabbits in comparison to both, Zemplin (*p* < 0.01) as well as New Zealand rabbits (*p* < 0.0001) (Table 2). An imbalance in the seminal oxidative milieu of specimens collected from Liptov Baldspotted rabbits was evident by a significantly decreased antioxidant capacity of the seminal plasma (*p* < 0.05 in comparison to New Zealand rabbits). Accordingly, a significantly increased oxidative damage to the sperm proteins (*p* < 0.05) and lipids (*p* < 0.001 in case of the Zemplin breed; *p* < 0.0001 with respect to the New Zealand breed) were observed in semen obtained from the Liptov Baldspotted rabbit breed.

**Table 2.** Oxidative profile of semen collected from three rabbit breeds.

Quality groups	Zemplin	Liptov Baldspotted	New Zealand
Reactive oxygen species production [RLU/s/10^6^ sperm]	2.91 ± 0.38	4.64 ± 0.77^**Z^	1.81 ± 0.31^****LB^
Total antioxidant capacity [eq. µmol Trolox/g protein]	9.98 ± 1.97	6.38 ± 1.37	13.72 ± 1.41^*LB^
Protein oxidation [nmol PC/mg protein]	2.15 ± 0.32	3.44 ± 0.43^*Z^	2.10 ± 0.22^*LB^
Lipid peroxidation [µmol MDA/mg protein]	2.51 ± 0.26	4.36 ± 0.52^***Z^	2.15 ± 0.29^****LB^

Mean ± S.D. *p < 0.05; **p < 0.01; ***p < 0.001; ^****^p < 0.0001.

^Z^–*vs*. Zemplin rabbits; ^LB^–*vs*. Liptov Baldspotted rabbits.

RLU: relative light units; PC: protein carbonyls; MDA: malondialdehyde

All analyses were run in triplicate.

### Inflammatory characteristics

The collected data reveal that the highest concentration of all pro-inflammatory factors was recorded in semen collected from Liptov Baldspotted rabbits (Table 3). While no significant differences were recorded in the case of CRP and IL-6, significant differences particularly amongst the Liptov Baldspotted breed and the New Zealand breed were detected in the case of leukocyte concentration (*p* < 0.01), the levels of IL-1β (*p* < 0.01) and TNF-α (*p* < 0.01). At the same time, significantly higher levels of INF-γ were recorded in ejaculates obtained from Liptov Baldspotted rabbits in comparison to the Zemplin as well as New Zealand breed (*p* < 0.05).

**Table 3.** Inflammatory characteristics of semen collected from three rabbit breeds.

Quality groups	Zemplin	Liptov Baldspotted	New Zealand
Leukocyte concentration [×10^6^/mL]	2.08 ± 0.56	3.44 ± 0.53	1.12 ± 0.24^**LB^
CRP levels [mg/dL]	0.72 ± 0.06	0.81 ± 0.07	0.70 ± 0.05
IL-1β levels [pg/mL]	4.26 ± 0.48	5.26 ± 0.63	3.51 ± 0.41^**LB^
IL-6 levels [pg/mL]	0.58 ± 0.14	0.64 ± 0.17	0.45 ± 0.12
TNF-α levels [pg/mL]	2.99 ± 0.32	4.09 ± 0.38	2.19 ± 0.29^**LB^
IFN-γ levels [pg/mL]	2.36 ± 0.36	4.34 ± 0.68^*Z^	1.96 ± 0.39^*LB^

Mean ± S.D. *p < 0.05; **p < 0.01.

^Z^–*vs*. Zemplin rabbits; ^LB^–*vs.* Liptov Baldspotted rabbits.

CRP: C-reactive protein; IL-1β: interleukin-1 beta; IL-6: interleukin-6; TNF-α: tumour necrosis factor alpha; IFN-γ: interferon gamma

All analyses were run in triplicate.

### Western blot

**Figure 1.**
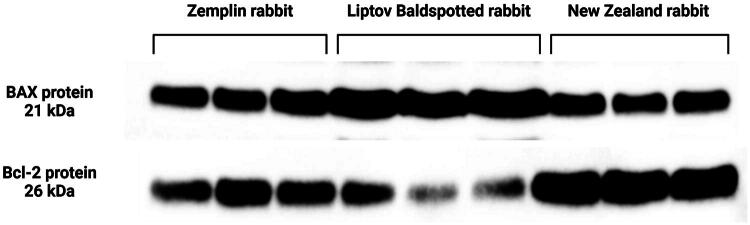
Expression patterns of the BAX and bcl-2 protein in spermatozoa collected from the analysed rabbit breeds. Photographs of the gel and original blots may be found as Supplementary Figures 1–3.

**Figure 2.**
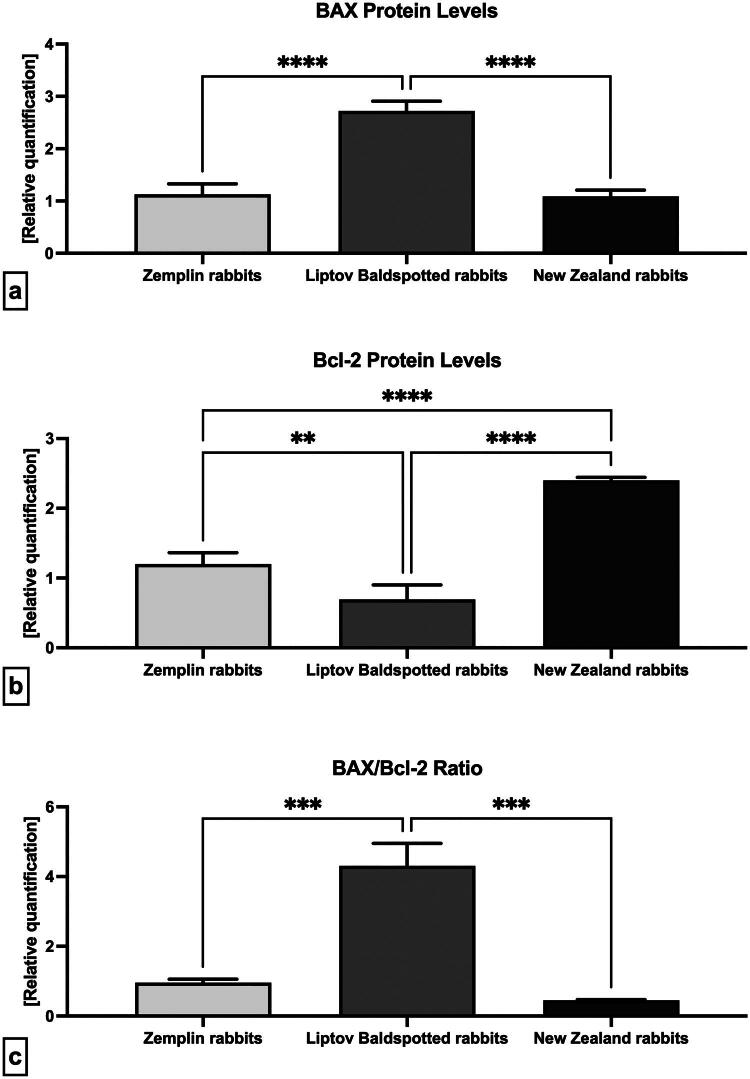
Graphical representation of the relative quantification of the (a) BAX and (b) Bcl-2 protein, as well as the BAX/bcl-2 ratio (c) in the analysed rabbit breeds.

Analysis of the selected proteins involved in apoptosis revealed significantly higher levels of BAX in spermatozoa collected from Lipov Baldspotted breed in comparison with both the Zemplin as well as New Zealand breed (*p* < 0.0001; Figs. 1 and 2(a)). This phenomenon was accompanied by significantly lower levels of Bcl-2 in the Liptov Baldspotted group when compared to the remaining breeds (*p* < 0.0001 in comparison with Zemplin rabbits; *p* < 0.01 against New Zealand rabbits; Figs. 1 and 2(b)). Accordingly, the BAX/Bcl-2 ratio was significantly increased in Liptov Baldspotted rabbit semen when compared to specimens collected from Zemplin rabbits as well as New Zealand rabbits (*p* < 0.001; Fig. 2(c)).

### Seminal plasma biochemistry

Analysis of the mineral profile of the seminal plasma revealed that the lowest concentration of Ca, Mg and P (Table 4) was found in the seminal plasma collected from Liptov Baldspotted rabbits, although no significant differences were found among the studied groups.

**Table 4.** Seminal plasma biochemistry amongst three rabbit breeds.

Quality groups	Zemplin	Liptov Baldspotted	New Zealand
Calcium [mmol/L]	2.88 ± 0.47	2.61 ± 0.33	3.97 ± 0.62
Magnesium [mmol/L]	6.52 ± 0.44	5.93 ± 0.58	6.57 ± 0.51
Phosphorus [mmol/L]	0.97 ± 0.19	0.50 ± 0.16	1.17 ± 0.29
Triglycerides [mmol/L]	1.03 ± 0.11	0.95 ± 0.18	1.09 ± 0.17
Cholesterol [mmol/L]	0.89 ± 0.13	0.75 ± 0.11	1.08 ± 0.12
Total proteins [g/L]	20.39 ± 3.41	20.04 ± 3.45	23.81 ± 2.54
Albumin [g/L]	7.57 ± 1.41	6.81 ± 1.99	7.73 ± 1.39
Uric acid [µmol/L]	42.29 ± 5.35	29.84 ± 4.55	57.62 ± 7.14
Urea [µmol/L]	0.20 ± 0.03	0.26 ± 0.05	0.12 ± 0.02
Alanine transaminase [U/L]	13.48 ± 2.11	9.82 ± 0.78^**Z^	15.48 ± 2.20^***LB^
Creatinine [µmol/L]	311.80 ± 28.53	188.50 ± 10.26^***Z^	451.60 ± 30.82^***Z; ****LB^

Mean ± S.D. **p < 0.01; ***p < 0.001; ^****^p < 0.0001.

^Z^–*vs.* Zemplin rabbits; ^LB^–*vs*. Liptov Baldspotted rabbits.

All analyses were run in triplicate.

Similar non-significant results were unravelled by the analysis of the triglyceride and cholesterol content, although the lowest levels of both parameters were found in the seminal plasma obtained from the Liptov Baldspotted breed.

In case of the seminal plasma protein profile, the lowest levels of total proteins, albumin and UA were found in specimens obtained from Liptov Baldspotted rabbits, however no significant differences among the breeds were recorded. Inversely, the highest urea concentration was observed in Liptov Baldspotted rabbit semen. While ALT levels were significantly lower in specimens obtained from Liptov Baldspotted rabbits when compared to Zemplin (*p* < 0.01) and New Zealand rabbits (*p* < 0.001), Crea concentration was significantly elevated in the seminal plasma obtained from Liptov Baldspotted breed in comparison with Zemplin (*p* < 0.001) and New Zealand rabbits (*p* < 0.0001). Significant differences were also detected among specimens obtained from the Zemplin breed and New Zealand breed (*p* < 0.001).

### Bacteriology

Using MALDI TOF mass spectrometry, 10 families, 11 genera and 21 bacterial species were identified in specimens collected from the Zemplin rabbit breed, out of which 57% were Gram-positive (G^+^) while 43% were Gram-negative (G^-^) bacteria. *Pseudomonas extremorientalis*, *Staphylococcus sciuri* and *Stenotrophomonas maltophilia* occurred more frequently in comparison to other bacterial species (Fig. 3(a)). In the case of the Liptov Baldspotted breed, 9 families, 10 genera and 15 species were isolates with 55% G^+^ bacteria and 45 G^-^ bacteria. Out of these, *Staphylococcus equorum* and *Stenotrophomonas maltophilia* were the predominant species isolated (Fig. 3(b)). Eight families, 9 genera and 14 species were found in ejaculates collected from New Zealand rabbits. In this case, 51% were G^-^ while 49% were G^+^ bacteria. The most frequent bacterial species found were *Stenotrophomonas maltophilia* and *Streptococcus salivarius* (Fig. 3(c)).

Figure 3.(a–c) Krona charts of the bacteria recovered from (a) zemplin, (b) Liptov Baldspotted and (c) New Zealand rabbit semen (outermost ring: species, Middle ring: genus, innermost ring: family).

**Figure:**
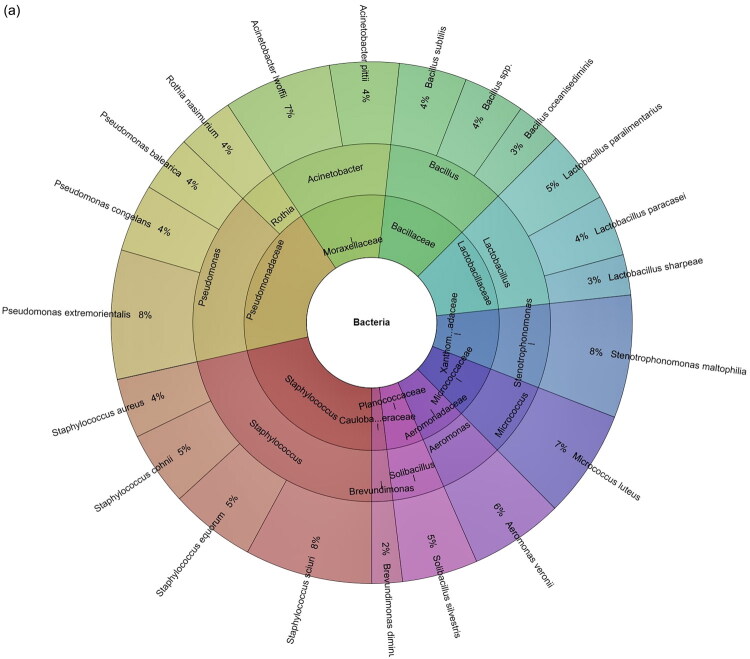

**Figure:**
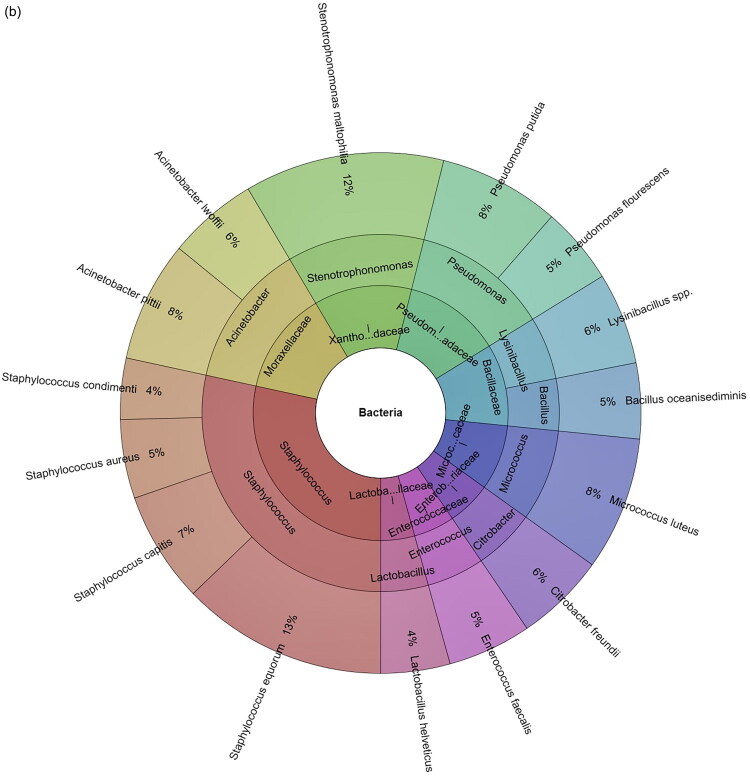

**Figure:**
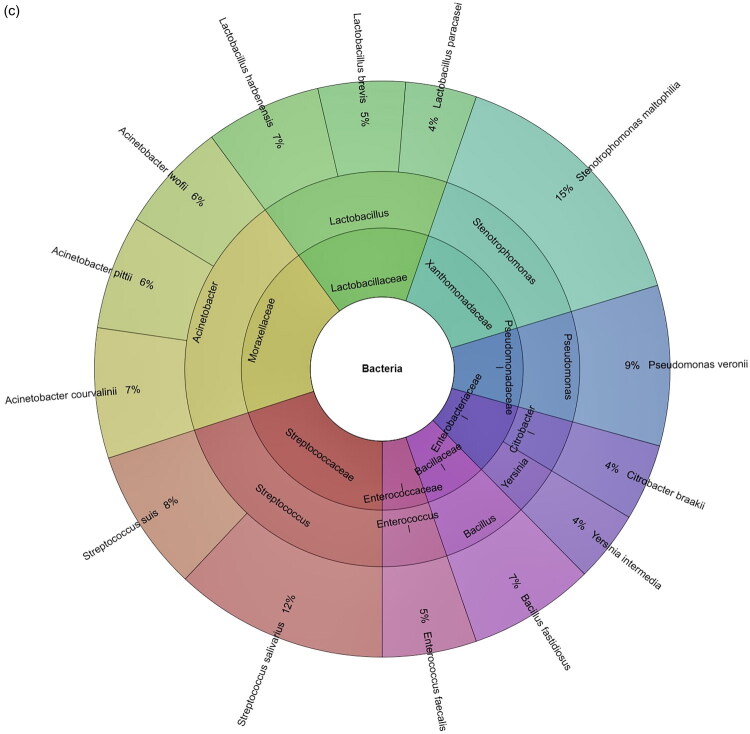

Randomly selected bacterial isolates with clinical relevance were tested for antimicrobial resistance (Supplementary Table 1) against amikacin (AMK), cefepime (CEF), chloramphenicol (C), ciprofloxacin (CIP), doripenem (D), gentamicin (GEN), imipenem (IMP), linezolid (LIN), meropenem (ME), tetracycline (TET), tigecycline (TIG), tobramycin (TOB) and vancomycin (VAN). The resulting inhibition zones were evaluated according to the breakpoint tables for interpretation of MICs and zone diameters version 13.0 (Sigma-Aldrich, St. Louis, MO, USA) (valid from 01/01/2023) issued by EUCAST (European Committee on Antimicrobial Susceptibility Testing). Intermediate resistance to linezolid was detected in case of the *Acinetobacter lwoffi*, *Acinetobacter pittii* and *Pseudomonas putida* isolates while the *Bacillus oceanisediminis* isolate was resistant to the antibiotic. While the *Bacillus cereus* isolate was resistant to tetracycline, intermediate resistance of the *Pseudomonas putida* isolate was detected in case of tigecycline, tetracycline and tobramycin.

The highest bacterial load (Supplementary Table 2) was recorded in semen collected from Liptov Baldspotted rabbits, although no significant differences were recorded among the breeds. Specific bacterial species retrieved from the ejaculates as well as sample prevalence of each bacterial isolate are listed in Supplementary Table 2.

### Biodiversity

A total of 16 different bacterial species were found in samples of Liptov Baldspotted breed. All of them were unique in their counts except for *Stenotrophonomonas maltophilia* with a total occurrence in three samples. Twenty-one different bacterial species were found in semen samples of Zemplin rabbit. Here, four bacterial species were represented by 8% and all the others were unique. A similar situation was for New Zealand White rabbits, where 16 different bacterial species were identified and *Stenotrophonomonas maltophilia* was the most abundant with the presence in 80% of samples.

A very distinctive bacterial profile was obtained in one of the analysed samples of Liptov Baldspotted rabbit with detected bacterial profile that contained *Lysinibacillus* spp., *Bacillus oceanisediminis*, *Solibacillus silvestris*, *Staphylococcus condimenti* and *Staphylococcus aureus*. In the samples of Zemplin rabbit, 60% of the analysed samples had very different profiles.

Comparison of the identified bacterial species showed distinct differences among the analysed rabbit breeds. A very specific bacterial community was identified in every individual breed. Five bacterial species were found in two of the compared breeds, most of the species present were specific for each breed. Only three species were present in all three analysed rabbit breeds–*Stenotrophonomonas maltophilia*, *Acinetobacter pittii* and *Acinetobacter lwoffii*.

Based on the obtained diversity indices, the richness of bacterial species was found to be highest in Zemplin rabbit. All values of the diversity indices were minimal in the analysed breeds. The Berger–Parker Index was low in all groups with the lowest values observed in the Zemplin rabbit what corresponds to a small domination of individual bacterial species throughout the analysed samples. The values of all indexes were affected by a small abundance and numbers of bacteria that were present in the analysed semen specimens (Supplementary Table 3).

## Discussion

The primary aim of this study was to assess the semen quality of three rabbit breeds in a larger context of conventional and non-conventional characteristics that may have an impact on the ability of spermatozoa to successfully accomplish fertilization. Our data reveal that New Zealand rabbits produced semen of the best quality in terms of sperm traits that are traditionally used to evaluate male fertility.^8^ Better sperm characteristics in New Zealand males could be attributed to their faster growth rate and earlier pituitary maturity in comparison with Zemplin and Liptov Baldspotted rabbits.^6^ This phenomenon could then positively affect the secretion of the luteinizing hormone (LH) and testosterone which will affect the maturity of semen.^34^ A superiority of New Zealand rabbits in comparison to traditional Egyptian Baladi and Sinai breeds was also reported by Abd El-Azim A and El-Kamash^35^ Similar high semen quality of New Zealand rabbits was found by Jimoh and Ewuola^36^ who compared the reproductive performance of four rabbit breeds under different mating systems, although superior semen characteristics were not necessarily correlated to the best overall fertility and litter sizes. On the other hand, both native Slovak breeds presented with a semen quality that rendered the samples as unsuitable for fertilization, particularly in terms of the sperm motility, which according to Lavara et al.^37^ should be higher than 70%. Hence, we may suggest that any eventual insemination doses of Zemplin and Liptov Baldspotted rabbits may be stored for later use only following appropriate sperm selection techniques.

Earlier reports on seminal bacteriomes have suggested that the sperm quality depends on the diversity of bacterial species as well as the quantity of bacteria present in the ejaculate, defined as the bacterial load.^13^^,^^17^ In the case of the bacterial load, as opposed to studies on larger farm animals, no significant variations in the total count of the bacterial colonies were found among the breeds. While it would seem plausible to hypothesize that in any highly specialized breed subjected to a greater selection pressure, the primary burden would be placed on the inherent defence mechanisms designed to identify and dispose of any potential pathogens,^38^ our results revealed that there were no differences among the studied rabbit breeds for the susceptibility of semen to contain bacteria. Nevertheless, a similar bacterial load was observed in previous studies on rabbits, primarily based on traditional bacteriological screening and identification techniques.^23^^,^^24^^,^^39^ On the other hand, notable differences were observed in the bacterial diversity among the breeds. Genera such as *Acinetobacter* spp., *Bacillus* spp. and *Pseudomonas* spp. were identified in all breeds with *Enterococcus* spp., *Micrococcus* spp. and *Staphylococcus* spp. being present in two out of three breeds, which agrees with earlier studies.^23^^,^^24^^,^^39^ Nevertheless, a greater prevalence of staphylococci and micrococci was observed in the ejaculates collected from the Zemplin and Liptov Baldspotted breed. In the meantime, a relatively high prevalence of lactobacilli was found in New Zealand rabbit semen. These notable differences are subject to speculation since all animals included in this study were of similar age and kept under identical conditions. Although the total bacterial load did not significantly differ among the rabbit breeds, this finding does not rule out a potential impact on semen quality. Instead, our results suggest that the composition and diversity of the bacterial community, rather than the absolute quantity of bacteria, may play a more decisive role in determining sperm functionality and inflammatory responses. Little to no information is currently available with on the unusual bacterial species found in the semen specimens of all breeds; thus, their origin and any impact on the resulting sperm quality shall be subject of future research. Based on the collected data we may speculate that the semen quality was impacted more by the bacterial diversity as opposed to the bacterial load, since the lowest sperm quality was found in semen collected from The Liptov Baldspotted breed, which contained more potentially uropathogenic bacteria, in particular *Staphylococcus aureus*. On the other hand, lactobacilli, predominantly found in New Zealand rabbits, have been suggested to act as an effective probiotic supplement for rabbits with potentially beneficial effects on the semen quality.^40^

At the same time, this study has unravelled antibiotic resistance patterns of several bacterial isolates retrieved from rabbit ejaculates, which is in line with recent evidence indicating a rising bacterial tolerance, or even resistance to antibiotics used in animal production. While data on the antibiotic susceptibility of bacteria retrieved from rabbit semen is very sparse,^41^ have unravelled increasing resistance patterns of bacterial colonies isolated from rabbit ejaculates to penicillin G, lincomycin and tylosin. As such, we may speculate that, in addition to the spread of antibiotic resistance in animal production and amongst consumers, the presence of bacteria irresponsive to antibiotics may cause further damage to male reproductive structures, and accelerate inflammation, as indicated by the cytokine assays in this study. The presence of antibiotic-resistant bacterial strains in rabbit semen raises important concerns for AI programs and overall breeding safety. The use of semen contaminated with resistant bacteria may compromise reproductive success by promoting subclinical infections, inflammation of the female reproductive tract, or a reduction in post-insemination sperm viability. Furthermore, a routine inclusion of antibiotics in semen extenders with the intention to control eventual contamination, may contribute to the incidence of resistant strains, especially in environments with a repeated exposure. This highlights the need for more targeted bacteriological screening of semen samples prior to AI, as well as the exploration of alternative antimicrobial strategies that are both effective and sustainable.^42^ Addressing resistance patterns in semen microbiota is therefore not only a microbiological concern but a critical component of a responsible reproductive management in rabbit breeding.

Immunity is inherently designed to counteract infection by releasing leukocytes to the source of inflammation. While under physiological circumstances white blood cells are in charge for the removal of dead spermatozoa, their overactivation may lead to phagocytosis of even healthy and viable sperm cells.^43^^,^^44^ Higher occurrence of leukocytes in ejaculates collected particularly from Liptov Baldspotted rabbits in this study agrees with earlier reports^13^^,^^17^^,^^25^ that postulate that leukocytospermia may compromise the lipid symmetry of the plasma membranes, even in otherwise healthy males. Activated immune response will also promote the release of cytokines, which may exhibit spermatotoxic properties with detrimental effects on the sperm proteins, lipids, and DNA^45^ and followed by apoptosis, as observed in this study. Within a large group of pro-inflammatory molecules, TNF-α which is predominantly secreted during infection, may promote sperm phosphatidylserine translocation and subsequent cell death.^46–48^ Moreover, IL-1 and IL-6 also seem to be involved in sperm deterioration, which corresponds with their increasing levels proportionately to a decline of semen quality as observed in this study as well as in previous reports on stud animals.^17^^,^^25^^,^^46^ High cytokine levels have been also reported to be correlated with oxidative stress,^17^^,^^25^^,^^45^ mitochondrial rupture and sperm motility inhibition,^17^^,^^49^^,^^50^ which is also corroborated by our data. Interestingly enough, our data collected from the cytokine assays may also provide us with a hint as to how semen will defend itself against bacterial contamination. Since bacteriospermia may stem from both internal and external sources, changes in the levels of pro-inflammatory molecules observed amongst the breeds may represent an adaptation phenomenon to physiological bacteriospermia or a defence mechanism against bacterial contamination post-semen collection. Nevertheless, the causes and reasons for such dynamic in the cytokine network are yet unknown and shall be elucidated in future studies.

Conjointly with inflammation, high ROS levels released by spermatozoa, leukocytes, aerobic as well as facultative anaerobic bacteria,^13^^,^^23^ (Schulte et al., 2019) may contribute to the onset and/or progression of oxidative damage to spermatozoa. Proportionately to elevated ROS levels, a significantly increased damage to the sperm proteins and lipids were detected particularly in ejaculates obtained from Liptov Baldspotted rabbits. Oxidative insults to the lipid bilayer of membranes, may endanger the semipermeable properties of the sperm surface. Our data also agree with previously published studies suggesting that cell death could be intricately involved in the promotion of ROS-inflicted sperm DNA fragmentation.^51^ Correspondingly, higher occurrence of spermatozoa with distortions to the membrane integrity and mitochondrial activity was accompanied by sperm chromatin disintegration in the semen specimens which has been frequently observed in suboptimal semen specimens.^49^^,^^52^

Bacterial contamination of semen often triggers an interlinked cascade of inflammatory and oxidative stress responses that can impair sperm functionality. Bacterial components, such as lipopolysaccharides (LPS), activate pattern-recognition receptors (e.g., TLR2 and TLR4) on male reproductive tract cells, initiating NF-κB-mediated signalling and the release of pro-inflammatory cytokines (IL-6 and TNF-α). Activated leukocytes exacerbate this response by producing large quantities of ROS, leading to lipid peroxidation, protein oxidation, mitochondrial damage, DNA fragmentation, and ultimately compromised sperm motility and viability (reviewed by Tvrdá et al.^53^) This suggests that even when overall bacterial loads are not significantly different, the presence and type of bacteria may be sufficient to disturb the delicate redox balance and incite localized inflammation that undermines semen quality.

Another important aspect of this study lies in changes observed in the seminal plasma of the studied breeds. Since reports on the roles of the seminal plasma biochemistry on male fertility are very sparse, we are currently left to speculate how specific seminal plasma components affect the resulting sperm vitality or the susceptibility of the ejaculate towards bacterial contamination. Our collected data reveal that the decrease of ALP activity in the seminal plasma was characteristic for ejaculates with a lower proportion of motile spermatozoa which is agreement with studies on bulls^32^ and boars.^54^ In the meantime, lower levels of Ca and Mg were found in the seminal plasma of specimens collected from the Zemplin and New Zealand rabbits. This observation could be explained by the fact that both elements act as critical regulators of sperm motility,^55^ while an earlier *in vitro* report revealed that the levels of both minerals were significantly decreased in the presence of staphylococci.^56^ UA has been reported to assist in the maintenance of a proper sperm motility, viability, and morphology by neutralization of oxidizing and nitrating agents.^57^ Since low UA levels have been recorded in the samples most compromised by oxidative stress, we may hypothesize that UA most likely actively counteracts ROS overproduction. The specific composition and amount of seminal plasma proteins have been also shown to play an important role in the promotion of the sperm motility (Henricks et al., 1998), capacitation and fertilization.^58^ In this study, lower levels of seminal plasma TP were detected in the samples with a lower semen quality. Although it was previously postulated^59^ that the total seminal plasma protein content is not a sufficient marker to predict possible causes of male infertility, earlier studies have indicated that decreased seminal plasma protein concentrations were associated with low sperm motility.^32^

Despite intriguing outcomes of this study, me must acknowledge that the obtained data are limited by the sample size, particularly for a multifactorial analysis.^60^^,^^61^ This constraint was primarily due to the limited population size and national conservation status of the Zemplin and Liptov Baldspotted rabbit breeds. These breeds are genetically valuable and rare, and only a small number of sexually mature males of similar age and health status were available at the time of sampling. Similarly, while the age range of 1–1.5 years may introduce variability, we deliberately selected animals within this narrow window to maintain group homogeneity and minimize confounding age-related effects. Given these constraints, we believe the current dataset still provides meaningful insights into breed-specific differences in semen quality.^62^

To our knowledge, this study represents the first attempt to comprehensively describe and compare conventional, non-conventional, bacteriological, biochemical, immunological, and oxidative parameters in selected important Slovak rabbit breeds. While a handful of reports has studied standard semen parameters in several Slovak rabbit breeds with different outcomes^19^^,^^24^^,^^63^^,^^64^ the reason for a lower semen quality in the native breeds in comparison to the New Zealand control has not been unravelled yet. Nevertheless, it seems that the Zemplin, and especially the Liptov Baldspotted breed may present with an inherently lower semen quality accompanied by a higher vulnerability to internal or external factors that may affect the resulting semen quality. As such, it may be of benefit to perform a larger scale analysis of possible genetic, epigenetic, or proteomic markers that may play a role in variations of the semen quality in rabbit breeds on more robust sample sizes to reduce Type II errors of the obtained data.^60^ At the same time, while this study provides a comprehensive evaluation of semen quality across multiple parameters, it does not include direct fertility outcomes such as AI success or litter size. Future studies incorporating controlled insemination trials are essential to validate the functional fertility implications of the observed semen quality differences and to support their translation into the breeding practice.^65^^,^^66^

## Conclusion

As rabbit breeding directly depends upon the quality of semen, attention must be paid to all the factors that may potentially put the sperm structural integrity and functional activity in jeopardy during the collection, processing, and storage of ejaculates. Summarizing the outcomes of this study, several key findings may be conveyed. Semen is a complex bodily fluid holding a network of interactions that may ultimately affect the sperm quality. Besides conventional semen quality characteristics such as the sperm concentration, motility or membrane integrity, advanced markers of sperm structural integrity and functional activity, including the mitochondrial function or DNA fragmentation may offer a broader view on the sperm behaviour and survival in *ex vivo* conditions. An important aspect lies in the importance of the seminal bacteriome which acts as a dynamic entity, the load and diversity of which may significantly impact the semen properties. Besides, bacterial profiles were unique to each breed, and the prevalence of uropathogens has a more decisive impact on the resulting sperm quality, as opposed to the bacterial load. In addition, oxidative stress and inflammation seem to act as hallmarks of a decreased semen quality, which is why we emphasize the introduction of their basic assessment in semen prior to its further processing. On the other hand, the seminal plasma components seem to play important roles in the protection of sperm vitality while preventing bacteria-inflicted damage to male gametes, which is why an increased attention should be devoted to their dynamics. Finally, several bacterial isolates recovered from the ejaculates presented with antibiotic resistance, which supports the need for a more efficient bacteriological screening of semen used for reproductive technologies, as well as for the development of strategies to prevent the spread of bacterial resistance in animal breeding.

## Supplementary Material

Supplementary figure 3.jpg

Supplementary Figures.docx

Supplementary figure 1.jpg

Supplementary tables.docx

Supplementary figure 2.jpg
