# Supplementary figures and images for: Variations in conventional and non-conventional semen characteristics of selected rabbit breeds
Source: Anim Biotechnol. 2025 Aug 22;36(1):2548300. doi: 10.1080/10495398.2025.2548300 (PMC12674288; doi:10.1080/10495398.2025.2548300)

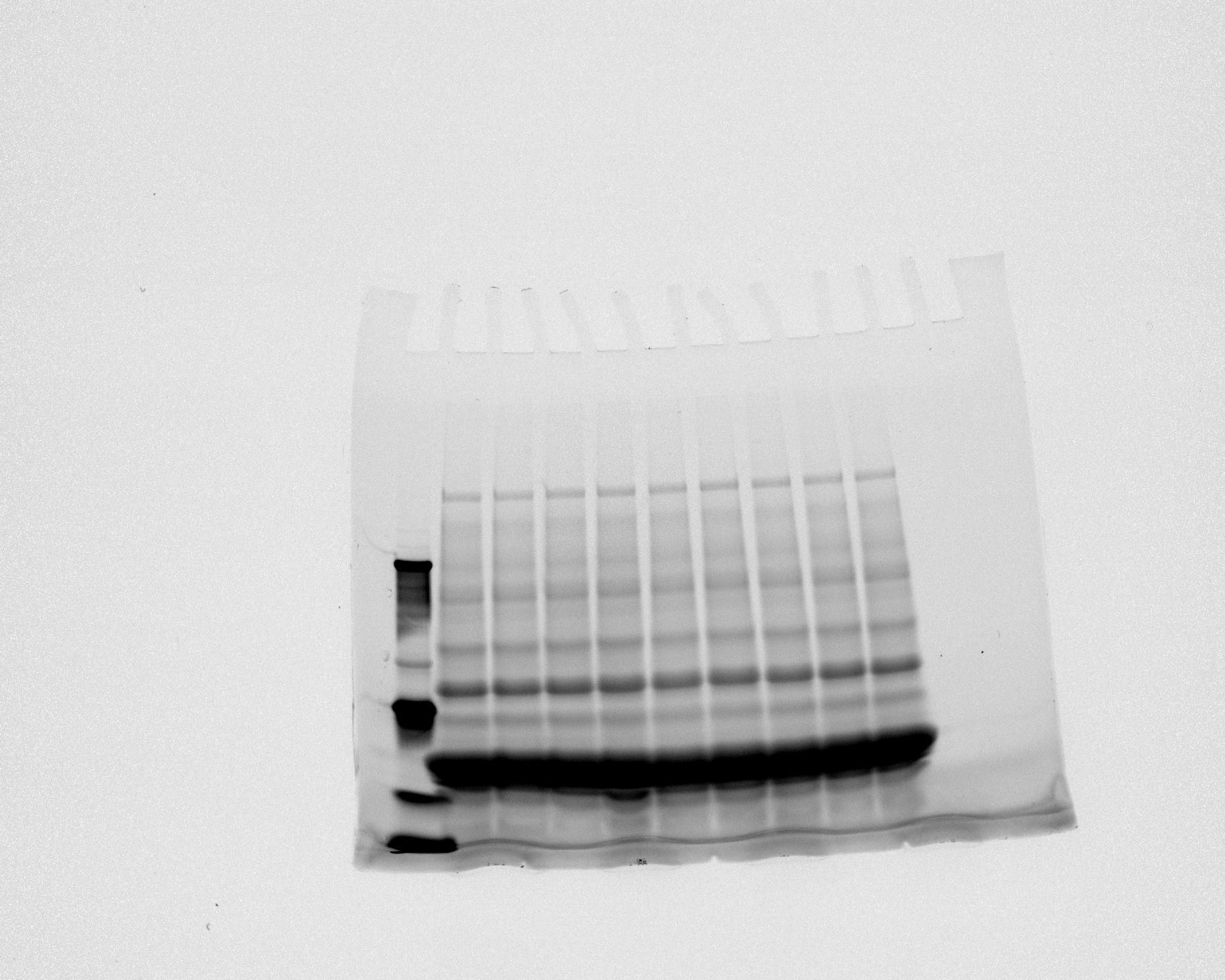

Supplement: Supplementary figure 3.jpg [file LABT_A_2548300_SM4861.jpg]

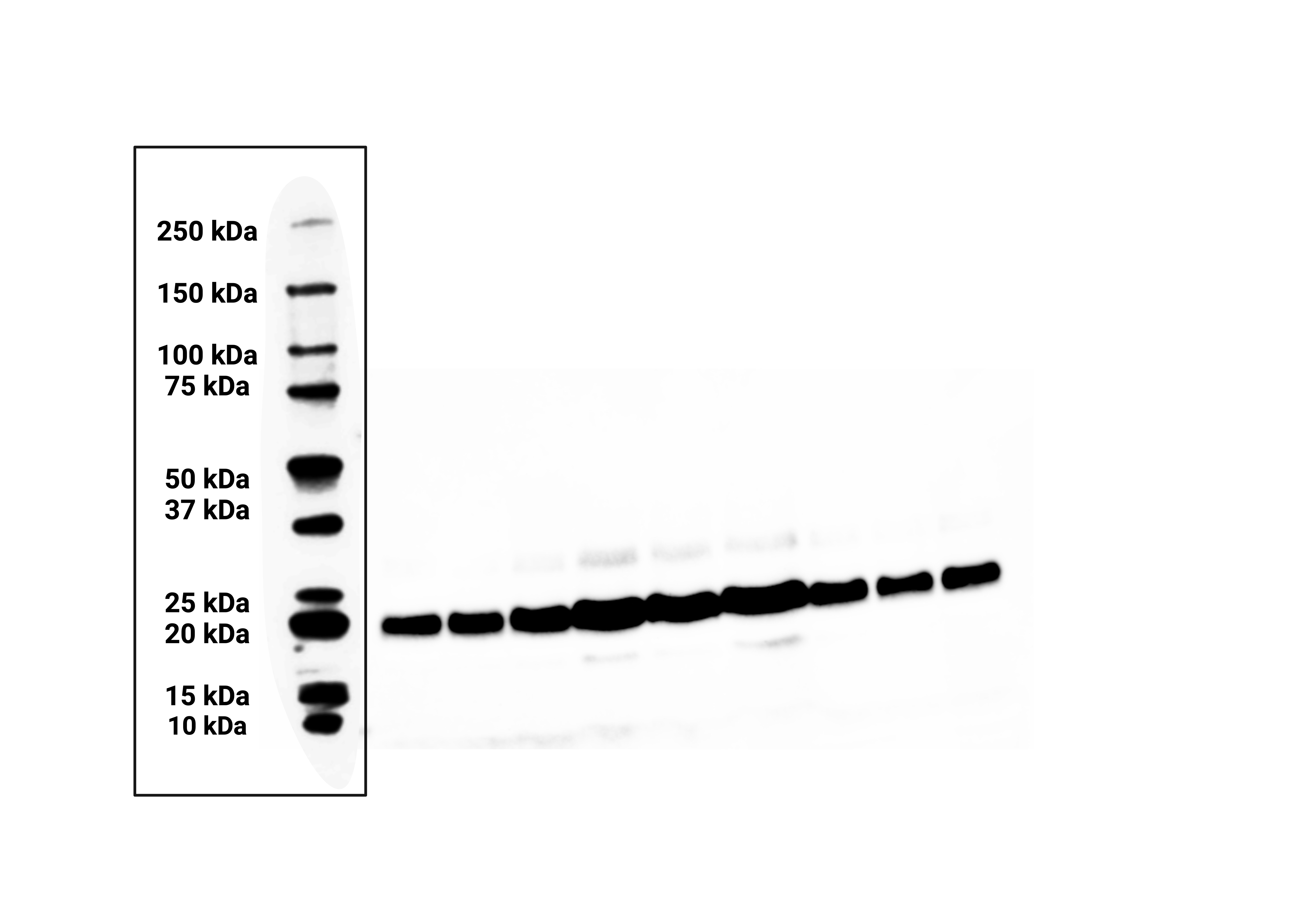

Supplement: Supplementary figure 1.jpg [file LABT_A_2548300_SM4859.jpg]

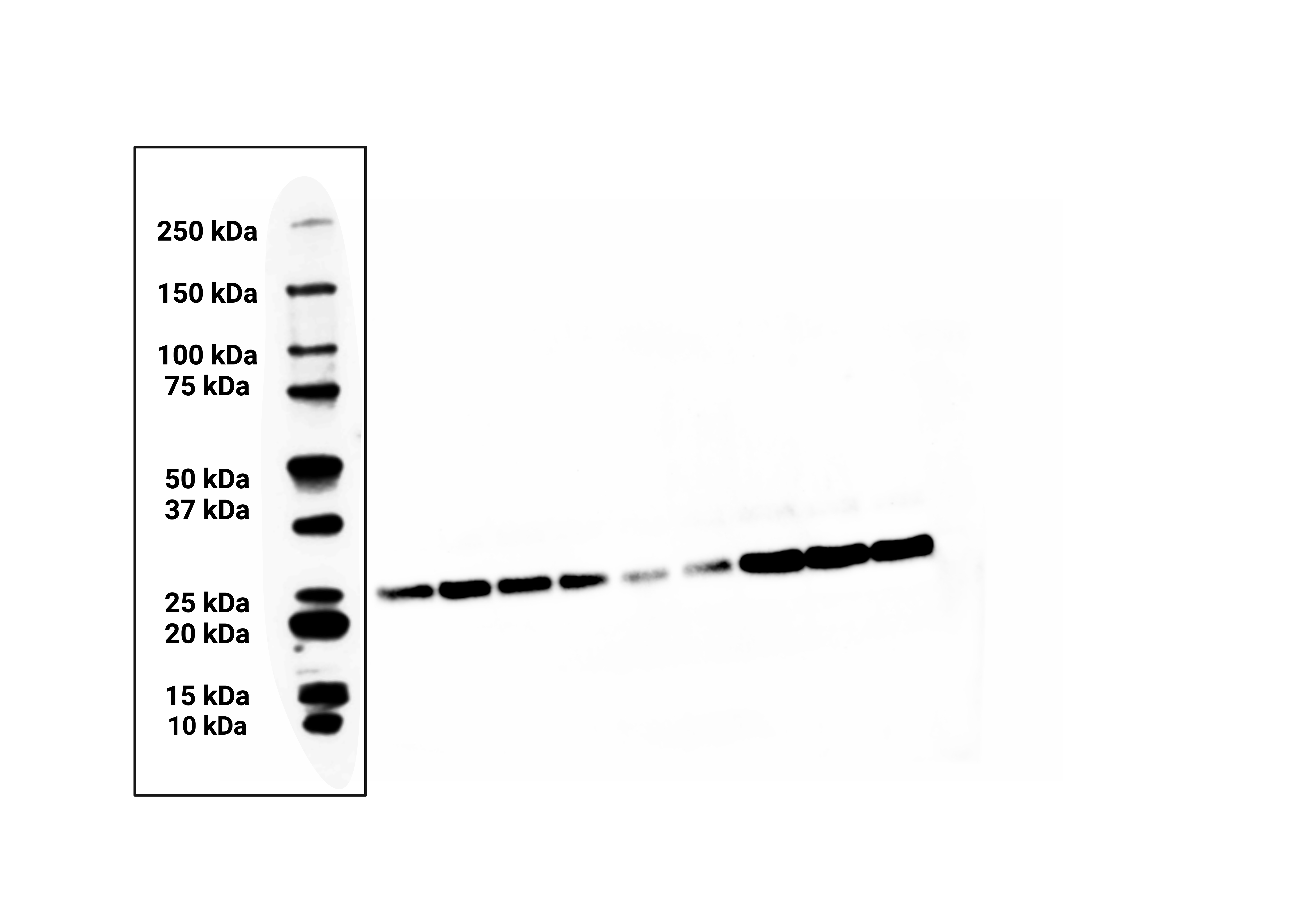

Supplement: Supplementary figure 2.jpg [file LABT_A_2548300_SM4857.jpg]
