# Supplementary material for: Variations in conventional and non-conventional semen characteristics of selected rabbit breeds
Source: Anim Biotechnol. 2025 Aug 22;36(1):2548300. doi: 10.1080/10495398.2025.2548300 (PMC12674288; doi:10.1080/10495398.2025.2548300)
Supplement: Supplementary Figures.docx [file LABT_A_2548300_SM4860.docx]

**Supplementary Figures**

SUPPLEMENTARY FIGURE 1 Original blot of the BAX protein.

SUPPLEMENTARY FIGURE 2 Original blot of the Bcl-2 protein.

SUPPLEMENTARY FIGURE 3 Representative photograph of the PAGE gel serving to confirm the loading uniformity.
