# Supplementary material for: Variations in conventional and non-conventional semen characteristics of selected rabbit breeds
Source: Anim Biotechnol. 2025 Aug 22;36(1):2548300. doi: 10.1080/10495398.2025.2548300 (PMC12674288; doi:10.1080/10495398.2025.2548300)
Supplement: Supplementary tables.docx [file LABT_A_2548300_SM4858.docx]

**Supplementary Tables**

SUPPLEMENTARY TABLE 1 Resistance profiles of bacteria recovered from rabbit semen samples.

| **Bacterium** | **AMK** | **CEF** | **C** | **CIP** | **D** | **GEN** | **IMP** | **LIN** | **ME** | **TET** | **TIG** | **TOB** | **VAN** |
| --- | --- | --- | --- | --- | --- | --- | --- | --- | --- | --- | --- | --- | --- |
| *Acinetobacter lwoffi* | S | ND | ND | ND | S | S | S | I | ND | S | ND | S | ND |
| *Acinetobacter pittii* | S | ND | ND | ND | S | S | S | I | ND | S | ND | S | ND |
| *Bacillus cereus* | ND | ND | ND | ND | ND | ND | S | S | S | R | ND | ND | S |
| *Bacillus oceanisediminis* | ND | ND | ND | ND | ND | ND | S | R | S | S | ND | ND | S |
| *Enterococcus faecalis* | ND | ND | S | ND | ND | ND | ND | S | S | I | ND | ND | ND |
| *Micrococcus luteus* | S | ND | S | S | S | ND | S | ND | S | S | S | S | ND |
| *Pseudomonas putida* | S | ND | ND | ND | ND | S | S | I | ND | I | I | I | ND |
| *Rothia nasimurium* | ND | ND | S | S | ND | ND | S | S | ND | S | S | S | S |
| *Solibacillus silvestris* | ND | ND | ND | ND | ND | ND | S | S | S | S | ND | ND | S |
| *Staphylococcus aureus* | S | S | S | S | S | S | ND | ND | ND | S | ND | ND | S |
| *Staphylococcus cohnii* | S | S | S | S | S | S | ND | ND | ND | S | ND | ND | S |
| *Staphylococcus epidermis* | S | S | S | S | S | S | ND | ND | ND | S | ND | ND | S |
| *Staphylococcus haemolyticus* | S | S | S | S | S | S | ND | ND | ND | S | ND | ND | S |
| *Staphylococcus simulans* | S | S | S | S | S | S | ND | ND | ND | S | ND | ND | S |
| *Staphylococcus succinus* | S | S | S | S | S | S | ND | ND | ND | S | ND | ND | S |
| *Stenotrophomonas maltophilia* | ND | ND | ND | ND | ND | ND | ND | S | ND | S | S | S | ND |

AMK - amikacin, CEF - cefepime, C - chloramphenicol, CIP - ciprofloxacin, D - doripenem, GEN - gentamicin, IMP - imipenem, LIN - linezolid, ME - meropenem, TET - tetracycline, TIG - tigecycline, TOB – tobramycin, VAN - vancomycin. ND – not defined, S – sensitive, I – intermediate, R – resistant.

SUPPLEMENTARY TABLE 2 Bacterial profiles of rabbit semen samples according to the breed.

| Groups | Bacterial species identified in semen and sample positivity | Bacterial load (log_10_ CFU/mL) |
| --- | --- | --- |
| Zemplin rabbit (n=10) | *Acinetobacter lwoffii* (60%), *Acinetobacter pittii* (20%), *Aeromonas veronii* (20%), *Bacillus oceanisediminis* (20%), *Bacillus subtilis* (40%), *Brevundimonas diminuta* (20%), *Lactobacillus paracasei* (20%), *Lactobacillus paralimentarius* (20%), *Lactobacillus sharpeae* (20%), *Micrococcus luteus* (40%), *Pseudomonas balearica* (40%), *Pseudomonas congelans* (40%), *Pseudomonas extremorientalis* (20%), *Rothia nasimurium* (20%), *Solibacillus silvestris* (20%), *Staphylococcus aureus* (20%), *Staphylococcus cohnii* (20%), *Staphylococcus equorum* (20%), *Staphylococcus sciuri* (20%), *Stenotrophonomonas maltophilis* (20%) | 2.78±0.61 |
| Liptov Baldspotted rabbit (n=10) | *Acinetobacter lwoffii* (20%), *Acinetobacter pittii* (20%), *Bacillus oceanisediminis* (20%), *Citrobacter freundii* (20%), *Enterococcus faecalis* (20%), *Lactobacillus helveticus* (20%), *Lysinibacillus fusiformis* (20%), *Micrococcus luteus* (20%), *Pseudomonas flourescens* (20%), *Pseudomonas putida* (20%), *Solibacillus silvestris* (20%), *Staphylococcus aureus* (20%), *Staphylococcus capitis* (20%), *Staphylococcus condimenti* (20%), *Staphylococcus equorum* (20%), *Stenotrophonomonas maltophilia* (60%) | 2.83±0.66 |
| New Zealand rabbit  (n=10) | *Acinetobacter courvalinii* (20%), *Acinetobacter lwofii* (20%), *Acinetobacter pittii* (20%), *Bacillus fastidiosus* (20%), *Citrobacter braakii* (20%), *Enterococcus faecalis* (20%), *Lactobacillus brevis* (20%), *Lactobacillus harbenensis* (20%), *Lactobacillus paracasei* (20%),  *Pseudomonas veronii* (20%), *Stenotrophomonas* *maltophilia* (80%), *Streptococcus salivarius* (20%), *Streptococcus suis* (40%), *Yersinia intermedia* (20%) | 2.70±0.59 |

SUPPLEMENTARY TABLE 3 Bacterial biodiversity characteristics of the analyzed rabbit breeds.

| Quality groups | Zemplin | Liptov Baldspotted | New Zealand |
| --- | --- | --- | --- |
| Richness (R) | 21 | 16 | 14 |
| Berger Parker Index Dominance Index | 0.08 | 0.17 | 0.22 |
| Shannon α-diversity | 0.03 | 0.03 | 0.03 |
| Simpson dominance | 0.05 | 0.07 | 0.09 |
